# Database of multi-parametric geophysical data from the TOMO-DEC experiment on Deception Island, Antarctica

**DOI:** 10.1038/sdata.2017.128

**Published:** 2017-09-12

**Authors:** Jesús M. Ibáñez, Alejandro Díaz-Moreno, Janire Prudencio, Daria Zandomeneghi, William Wilcock, Andrew Barclay, Javier Almendros, Carmen Benítez, Araceli García-Yeguas, Gerardo Alguacil

**Affiliations:** 1 Instituto Andaluz de Geofísica, University of Granada, Granada 18071, Spain; 2 School of Environmental Sciences, University of Liverpool, Liverpool L69 3GP, UK; 3 Department of Earth and Planetary Sciences, University of California, Berkeley, CA 94720, USA; 4 Independent Consulter, Forth Management LLP, Alpside Solutions, 16 High Street, Dalbeattie Dumfries & Galloway DG5 4AA, UK; 5 School of Oceanography, University of Washington, Box 357940, Seattle, WA 98195-7940, USA; 6 Lamont-Doherty Earth Observatory, Columbia University, Palisades, NY 10964, USA; 7 Department of Signal Theory, Telematics and Communications, University of Granada, Granada 18071, Spain; 8 Department of Applied Physics, University of Cádiz, Cadiz 11519, Spain

**Keywords:** Volcanology, Seismology, Geophysics

## Abstract

Deception Island volcano (Antarctica) is one of the most closely monitored and studied volcanoes on the region. In January 2005, a multi-parametric international experiment was conducted that encompassed both Deception Island and its surrounding waters. We performed this experiment from aboard the Spanish oceanographic vessel ‘Hespérides’, and from five land-based locations on Deception Island (the Spanish scientific Antarctic base ‘Gabriel de Castilla’ and four temporary camps). This experiment allowed us to record active seismic signals using a large network of seismic stations that were deployed both on land and on the seafloor. In addition, other geophysical data were acquired, including bathymetric high precision multi-beam data, and gravimetric and magnetic profiles. To date, the seismic and bathymetric data have been analysed but the magnetic and gravimetric data have not. We provide P-wave arrival-time picks and seismic tomography results in velocity and attenuation. In this manuscript, we describe the main characteristics of the experiment, the instruments, the data, and the repositories from which data and information can be obtained.

## Background & Summary

**Figure 1:**
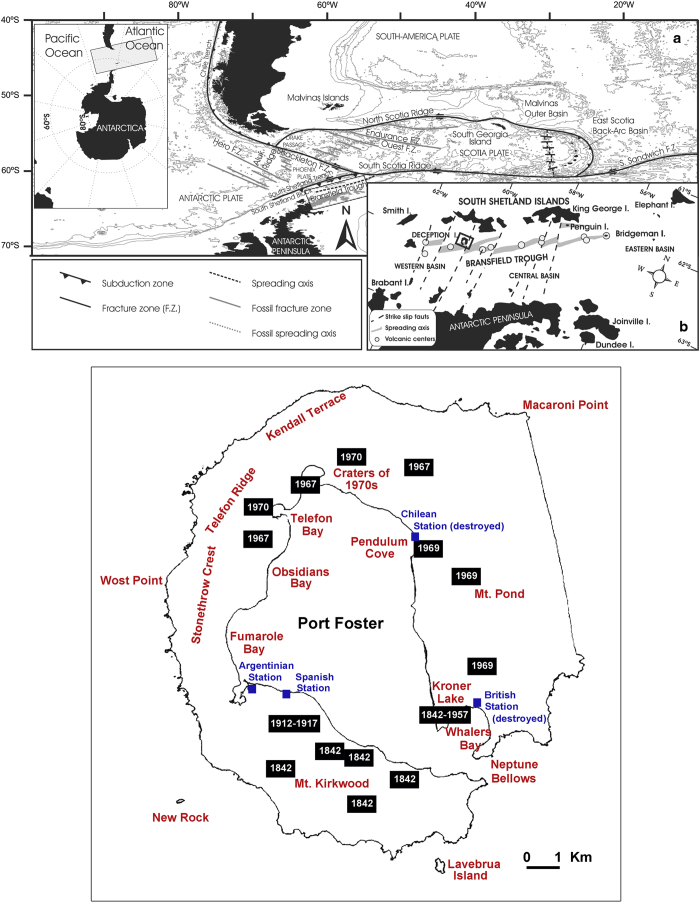
Maps of Deception Island showing the regional tectonic setting and locations of sites relevant to this study. (top) Location of Deception Island plotted in a regional framework and showing nearest plate boundaries and observed regional rift areas (**a**). The box shows the location of Deception Island (**b**). (bottom) The shape of Deception Island with the locations of recent volcanic eruptions (dates shown inside black boxes), the main scientific stations and bases (in blue), and some of the geographical sites cited in the text.

Multi-parametric geophysical experiments using marine and terrestrial techniques on active volcanoes are rare^1^. The TOMO-DEC experiment is a pioneering study that has combined several techniques to provide multi-disciplinary information that is more complete than previous studies at Vesuvius volcano^2^ and Campi Flegrei^3^, Italy. We performed this experiment at Deception Island volcano (Antarctica; Fig. 1) using the Spanish oceanographic research vessel ‘Hespérides’ as a platform for generating active seismic sources and as a recording system for other geophysical data, including magnetic and gravimetric fields. We recorded seismic signals using a temporary network of 95 land-based seismometers deployed on Deception Island and 14 ocean-bottom seismometers (OBS) deployed on the floor of a flooded caldera (Port Foster) and in the Bransfield Strait around the island. These included 7 Lennartz Marslite seismic stations, 4 M24 instruments, and 11 seismic arrays, all working in continuous recording mode. We generated active seismic sources using an air gun array with a maximum capacity of 3,520 cubic inches (c.i.). Together with the seismic waveforms, we collected a final dataset consisting of travel times for more than 70,000 crustal P-wave first arrivals. We recorded bathymetric data using both multi-beam and single-beam sounders. We obtained magnetic profiles using a Marine Magnetics SEASPY marine magnetometer on the base of an Overhauser sensor. Finally, we acquired gravimetric data using the marine gravimeter BELL AEROSPACE-TEXTRON BGM-3. In total, the various data types accounted for more than 1,000 km of profiles.

Deception Island (Antarctica) is an active volcano of considerable scientific interest. Its origin and evolution remains controversial; however, most recently it has been interpreted as a collapse caldera resulting from the explosive eruption of basaltic-to-andesitic magmas^4^. A reconstruction of paleo-volcanic geometries^5^ reveals an initial stratovolcano island that reached 640 m above sea level. The present caldera hosts a very active geothermal system, which is responsible for most of the present-day seismic activity^6–10^. The importance of research at Deception Island is widely accepted and a recent review of multidisciplinary research performed on the island^11^ showed that between 1964 and 2012 at least 173 impact papers have been published, with the number of new publications growing exponentially. This scientific interest is related to the continuous presence of multiple research teams, including those stationed at permanent bases (e.g., the Spanish Antarctic base ‘Gabriel de Castilla’ and the Argentinean base ‘Deception’), or in temporary camps. The large number of researchers working in the volcano is a clear increasing of the volcanic risk^12^. However, this increase in volcanic risk is primarily the result of increasing visitor numbers, with thousands of tourists accessing the island each year^13^.

In this manuscript, we present a dataset that has been analysed using different approaches and techniques and which has provided new insights into the nature and structure of the island. Tomographic and structural images of seismic velocity^14,15^ and attenuation^16,17^ have been used to help in the interpretation of the inner structure of the volcano, sometimes combined with bathymetric analysis^18^. The interpretation of these results indicates the presence of strong lateral structural variations that affect seismic wave propagation^19,20^. The presence of many volcano-clastic deposits also introduces near-surface low velocity effects^21^. This velocity heterogeneity can produce changes in the seismic-waveforms of the classic volcanic signals used to assess volcanic risk (e.g., LP events^22^). Finally, when analysing the data, researchers considered the impact of unique local weather conditions, in which warm water increases the presence of biological activity^23^.

Although considerable information has already been extracted from the dataset, there remains the potential for additional research given the high quality of the data, the volume of unanalysed (magnetic, gravity) or partially-analysed data (bathymetry), and the recent advances in the field of signal processing, from automatic signal recognition techniques^24–27^ to new methods for the determination of first arrivals^28–30^. We expect that our data is of broad interest and may yield new and high-impact results that will add to the existing scientific impact of the experiment. The database includes not only the raw data, but also seismological models, such as 3-D velocity and attenuation structures.

## Methods

Deception Island volcano is a remote location where complex experiments require a large number of people, expensive logistical complexities, and complex material support including transport and accommodation. The success of the TOMO-DEC experiment extends beyond the above-cited scientific and social implications. Moreover, the results have provided additional support to other multidisciplinary approaches, including those in deformation and geodesy^31,32^, magnetic and gravimetric models^33,34^, and other tomographic and structural studies^35–37^. On this basis, we believe that this multidisciplinary database must be opened to the scientific community. Finally, it is important to remark that we provide derived models associated with the structure of Deception Island, mainly 3-D velocity structure and attenuation models, which will be important for scholars wishing to apply new advanced relocation techniques, such as has been done in several volcanic environments^38^.

Despite the large number of geological and geophysical studies performed at Deception Island, existing volcanological models lack complete information on the inner structure of the island. This shortcoming was acutely felt during a seismo-volcanic crisis that occurred during the 1998–1999 Austral summer-autumn^8^. In the absence of realistic velocity and volcanological models, it was not possible to distinguish the real triggering process of this crisis and the nature (volcanic or tectonic) of the observed seismic stress migration. We conceived the TOMO-DEC experiment to improve our knowledge, and by implementing a robust interpretation of the results^9^, we were better able to inform the emergency and contingency volcanic eruption plan^12^. This experiment was organised in collaboration with several research institutions. For fieldwork and data collection, the following institutions participated (each providing seismic instruments and/or researchers): Instituto Andaluz De Geofísica, Spain (12 researchers, 10 type land seismic arrays); Lamont Doherty Earth Observatory, Columbia University, USA (4 researchers, 14 OBS); INGV-Osservatorio Vesuviano, Italy (1 researcher, 7 Marslite type land stations); INGV-Catania, Italy (1 researcher, 4 M24 type land stations); CENAPRED, Mexico (1 researcher); Universidad De Cádiz, Spain (2 researchers); Universidad De Colima, Mexico (1 researcher); University College Dublin, Ireland (1 researcher); Universidad Complutense De Madrid, Spain (1 researcher); Universidad De La Plata, Argentina (1 researcher); University of Washington, USA (3 researchers); and the USGS Volcanic Hazard Team, USA (2 researchers).

### TOMO-DEC experiment

The TOMO-DEC experiment was organised into three main stages: (1) a search for sites to deploy seismic stations and establish temporary camps to accommodate researchers during the austral summer of 2003–2004; (2) field-based data collection during the austral summer of 2004–2005; and finally (3) laboratory-based data gathering, organisation, and analysis. Stage 3 remains on going and papers continue to be published. The preliminary analysis of data involved an initial phase of data gathering from instruments of different types, followed by their organisation in a joint database, their conversion to a common format, and finally the determination of the first P-wave arrival times.

**Figure 2:**
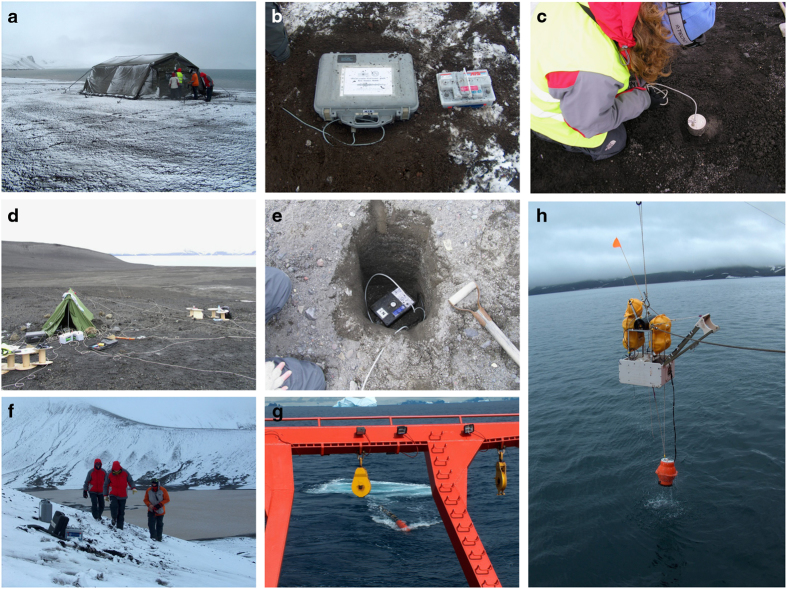
A set of images showing details and examples of seismic stations deployment, including ocean-bottom seismometers (OBSs) recovery and the air-gun operation. (**a**) An image showing the type and size of one of the temporary camps deployed during the TOMODEC experiment. (**b**) An example of an autonomous seismic stations deployed on permafrost, including the batteries used for power supply. Seismometers were buried as indicated in (**c**,**e**). The depth of each hole was controlled by permafrost conditions. The central hub of each seismic array was protected using tents as illustrated in (**d**). Since each seismometer was connected by cable to this hub, many thousands of metres of cable were used in the experiment. (**f**) An image illustrating the complex access of some of the autonomous seismic station. (**g**) Image of the bubble formed at the sea surface following an air gun shot from the ‘Hespérides’ vessel. (**h**) A picture illustrating the recovery of an OBS from inside of Port Foster.

Stage 1 was performed during two summer field expeditions (2002–2003 and 2003–2004). The whole island was surveyed (including glacial areas) in order to identify potential sites for seismic stations and camps. We assembled a handbook for use during the 2004–2005 summer deployment that included precise locations (by hand-held GPS) and cross-island access routes. We identified four sites for temporary camps (Whalers Bay, Pendulum Cove, Sea Wolf Cove, and Obsidian Valley; Fig. 1), each to house 6–8 researchers and include an adapted shelter tent, beds, food, and other necessities (Fig. 2). We identified 170 potential sites for seismic stations, including flat areas where seismic antennas could be deployed. This number was larger than the final number of sites used in the experiment.

Stage 2 (the experiment) took place between December 2004 and March 2005, with most of the activity focused on the deployment of seismic stations (on land and OBSs), air gun shooting, data collection, and the final recovery of the majority of the seismic stations. Data collection was concentrated between 4 January and 24 January 2005. At the end of the Antarctic season, some OBSs and on-land stations were deployed to record local seismicity. The experiment was completed on 1 March 2005.

### Geometry of the experiment and observations

In December 2004, we deployed four radio-telemetered seismic stations at different sites on the island (one 3-component station and three vertical-component stations) to monitor the background level of seismic activity. In January 2005, 11 individual seismic stations and 10 arrays with a maximum of 12 channels each were deployed. This work included the resurvey of pre-established station sites, their installation, and a status check. For the single stations, this check consisted of a true plug-and-play and a real-time check in the field (Fig. 2). The array installation comprised individual sensors connected to the central hub of the acquisition system by 50- to 200-m-long cables. Data could be checked in a quasi-real time after conversion to a readable format (Fig. 2). Each seismometer was buried as deeply as possible in what were typically pyroclastic deposits over permafrost. In most cases the burial depth was~20 cm, and the deepest hole was 50 cm (Fig. 2). In glaciated areas, holes were dug to depths sufficient to cover the sensor and minimise wind noise. The central hubs of the acquisition systems of many stations were protected inside tents (Fig. 2). The position of each seismic station was initially determined using hand-held GPSs; however, during the experiment, improved positions were obtained using the differential GPS system of the ‘Hespérides’ for both individual seismic stations and for the central hub of every seismic array. A laser theodolite was then used to measure the height of each array node with respect to the differential GPS solution for its central hub.

**Figure 3:**
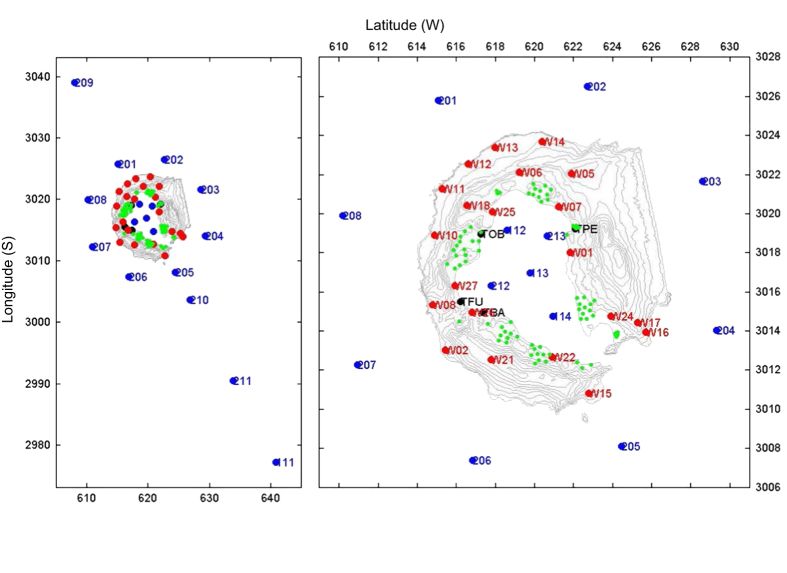
Maps showing the positions of seismic stations deployed during the experiment. Green denotes short period seismometers associated with array systems. Red denotes 3-component autonomous seismic stations. Black denotes telemetered seismic stations. Blue denotes ocean-bottom seismometers (OBSs). A file named stations_data_05022006.xls contains all station positions and is located in the ‘Seismic’ folder.

At the same time, a total of 14 OBSs were deployed. Of these, 11 were placed offshore of the island at depths between 200 and 686 m below sea level (b.s.l.), while 3 were placed inside Port Foster Bay at a maximum depth of 150 m. This procedure included on-deck instrument preparation, deployment on the seafloor, and acoustic verification of the operating status. The entire deployment of instruments took 3 days. During days 8 to 11, the first pattern of air gun shots was fired, both inside and outside the bay of Post Foster. After the first shooting phase, 10 of the autonomous seismic stations were relocated in order to increase the number of recording sites to 21. At the same time, 10 of the 14 OBSs were also recovered and redeployed at new locations. A second phase of air gun shooting was performed between 16 January and 18 January. The positions of all seismic stations deployed during the experiment are shown in Fig. 3. Following the second phase of air gun shooting, most of the seismic stations were recovered. Four M24-type land stations and 12 OBS were left in their final positions until late February 2005 in order to record natural seismicity in and around Deception Island volcano.

**Figure 4:**
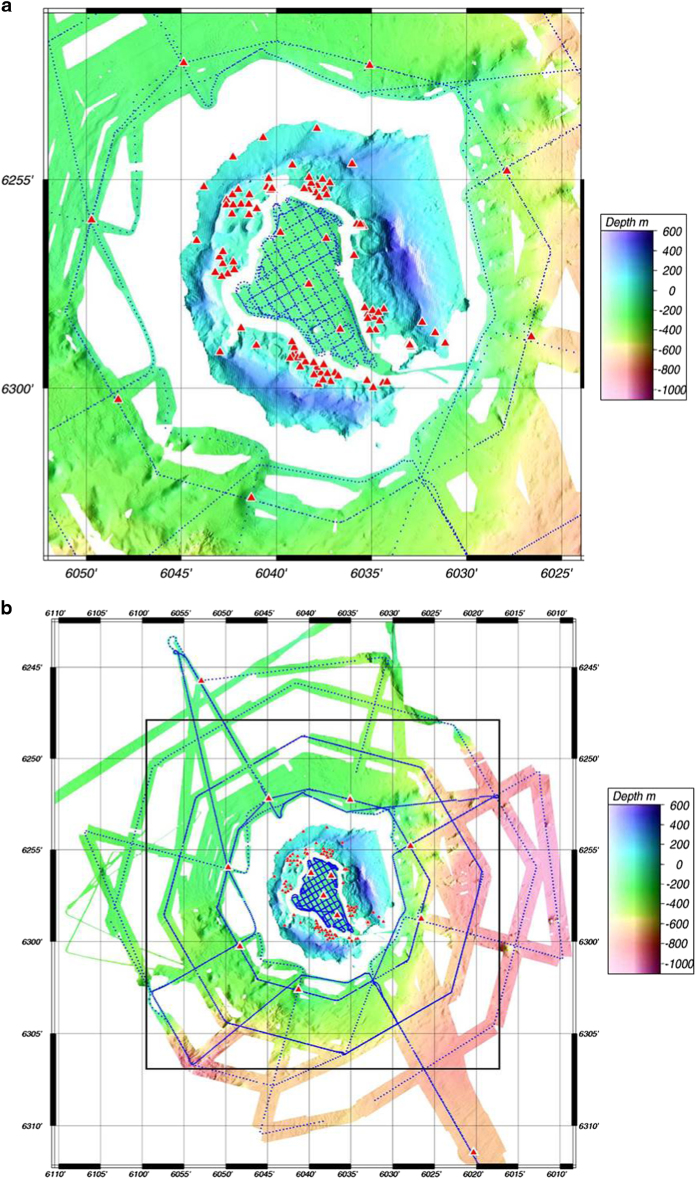
(**a) Path followed by the ‘Hespérides’ vessel inside of Port Foster (Deception Island) and in the nearest surrounded area on one of the shooting legs.** Red triangles represent the positon of the seismic stations. Blue dots are the position of every shot. In a color scale elevation and sea depth are indicated. For the sea depth we used the bathymetric data set provided in this manuscript. (**b**) Path followed by the ‘Hespérides’ vessel offshore of Deception Island on one of the shooting legs.

The active sources for the seismic experiment were generated using air guns towed behind the R/V ‘Hespérides’ of the Spanish Research Council, and conducted by the Spanish Army. This process was conducted by the Spanish Army. The air gun shot lines were designed to provide dense coverage inside and around the island. In the interior of Port Foster, the tracks followed a dense grid of perpendicular lines with a shot spacing of 120 m on a 0.5 km grid and with shots fired every 60 s (Fig. 4). A safety distance from the coast of~500 m was respected. Offshore of the island, shots were fired every 120 s at intervals of 170–340 m along three main trajectories (Fig. 4): (1) two straight tracks for refraction profiles, one 92 km long and oriented NNW-SSE and the other 55 km long and oriented WSW-ENE; (2) concentric lines around the island (out to 20 km from the centre of the island); and (3) lines in a 'star' configuration. The theoretical tracks were planned on the basis of station distribution, with the aim of sampling known tectonic structures with an optimum source and receiver configuration. For example, the seismic line oriented NNW-SSE was designed to obtain a 2-D section of the expected velocity contrasts NW of Deception Island^15^. For the inner part of the island (Port Foster), two rounds of shooting were performed. In the first round, only four guns were fired with a total capacity of 1520 c.i. (24.91 l or 0.024908 m^3^). In the second round inside the bay, the total capacity was increased from 1520 to 2020 c.i. (33.1 l or 0.033101 m^3^). Approximately 380 nautical miles (more than 700 km) of air gun shooting were collected. This configuration, along with the operational capability of the air guns allowed the recording of high-energy P-wave signals at distances as great as 40 km. The dataset consists of 6,630 useful shots.

While the ship was shooting, deploying, or recovering, additional geophysical and marine data were also recorded. In particular marine gravimetric data, magnetic measurements, and multi-beam eco-sounder signals (using two types of instruments) were collected.

### Active seismic source measurements

#### Seismic sources

**Figure 5:**
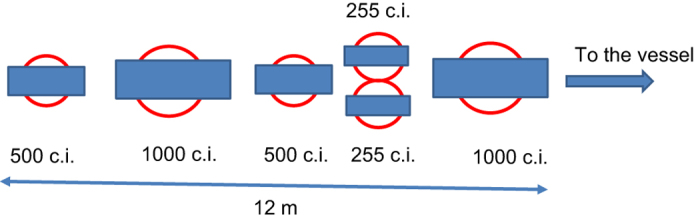
Image showing the air gun configuration (with size in cubic inches; c.i.) mounted on the ‘Hespérides’ vessel and used during the TOMODEC experiment to generate seismic signals. Circles denote the theoretical position of the highest energy bubble obtained with this array of air guns.

Active seismic sources were generated using six BOLT 1,500LL air guns in an array configuration 12-m long (Fig. 5) that was chosen to optimise the directivity of the source and minimise the water bubble. The bubble was generated using four Hamworthy 4TH190W70 compressors producing a maximum pressure of 140 bars. The air guns had capacities of 500, 1,000, 500, 255, 265, and 1,000 c.i., giving a total volume of 3,520 c.i. (57.7 l), and had inter-gun spacings of 0.8–2.5 m. The power of the system was decreased to 1,520 and 2,020 c.i. (as indicated above) for shallow-water shooting inside Port Foster. Each shot was recorded by a near-field hydrophone. The average of depth of the air gun array was 9 m b.s.l. In the link https://data.aad.gov.au/metadata/records/TOMODEC_2005_UTM-SPAIN we provide the whole information of the temporal and spectral characteristic of the air gun source, with the technical features of the air gun system detailed in Table 1. It is noteworthy that during the whole experiment no problems were detected and all of the planned shots were fired.

The air guns were controlled by a Hydra Minipulse system, which synchronised the guns and triggered the shots. Using information from a sensor located in each gun, the system can correct the moment of fire in order to synchronise shots to within 1 ms. The shot command is initially supplied by the GPS Seapath clock to the KONMAP navigation system (fix point) with a delay of 1 ms. This information is then sent to the gun control system within 60 ms. Summing up these time intervals, the real shot time has a delay of 61 ms with respect to the time written in the telegram supplied for every shot. This telegram contains information about the fix point (e.g., latitude, longitude, date, direction of vessel, speed, depth, name of the line of shots, and number of the fix point). The location information applies to the position acquired 60 ms earlier (after correction for the distance between the GPS and gun). After the shots, another message is appended to these data by the Minipulse system, detailing the number of working air guns, and the total capacity and delay of each gun with respect to the aiming point. All of these data are supplied for every shot and are recorded on a PC in text format. The whole telegram is made up of two parts: (1) data supplied by the Minipulse system (starting with the $HYDRA sequence), and (2) data from the navigation system ($HESSIS sequence). Owing to the huge amount of data, the output files were filtered in order to make them more manageable. Filtered files contain 6 initial fields (shot number, latitude, longitude, date, hour, fix), of which the first field was extracted from the shot telegram, while the others were extracted from the navigation telegram. In addition, a log file was supplied detailing system failures and errors. As a result of this organisation, the data appear as reported in Table 2.

The gun positions were assumed to be 125 m astern and 5 m to port of the GPS antenna, with a constant depth of 9 m. In the UTM conversion to geographical coordinates, the UTM region was 20E.

#### Seismic instruments

*Lennartz Marslite autonomous stations:* The 7 Marslite seismic stations used in the experiment were Lennartz LE-3Dlite MkII short-period sensors that record 125 samples per second using a 3-channel, 20-bit data acquisition system. This system includes a preamplifier with four 12 dB steps. Since digitisation always takes place at a fixed sampling rate of 4 kHz, the digital data stream needed to be decimated to the final sampling interval, after the signal had been digitally low-pass filtered by a FIR (Finite Impulse Response) filter. The system was completed with a GPSlite time signal receiver. The data were recorded in binary format on rewriteable 540 Mb magneto-optical disks. The acquisition systems were programmed to record in continuous mode. The power supply was provided from an external 12 V DC supply, later stabilised and regulated to the voltages required by the system.

*Lennartz M24 seismic stations:* Four of the stations used Lennartz M24 Compact/LP portable seismometers with 24 bits of dynamic range working with 3 built-in channels. There is one separate over sampled A/D converter per input channel, which digitises the signal coming from the preamplifier at very high frequency (20 kHz), until such point that the data stream is decimated down to the user's desired sampling rate: in our case, a sampling frequency of 100 Hz was configured. A single chip works as both a general-purpose CPU (Central Processing Unit) and as a DSP (Digital Signal Processor). The latter functionality is required to achieve the high-speed, high-precision digital filtering commonly associated with over sampled ADCs. GPS timing is performed thanks to a built-in receiver combined with an exterior antenna connected through a cable. The selected data format was of binary type, and recording was on a removable 2.5'' IDE 20 GB hard disk. For power supply, external 12 V DC power is accepted (in our case, a 100 A/h battery in combination with a 47 W solar panel) and converted to internally required voltages. The acquisition system is equipped with a Lennartz LE-3D (20 s) seismometer, which has a corner frequency of 0.05 Hz, an output sensitivity of 1,000 V/m/s, and damping of 0.707.

*Seismic arrays:* The seismic array modules were high resolution (24 nominal bits) with an acquisition system that can work with a maximum of 12 sensors, in array configuration. In this experiment, the acquisition modules were used with 3–12 channels (see below). Sampling frequency was configured to 100 samples/s and the gain was set to 1. Data acquisition was performed by four SEISAD18 plates, each managing three analog channels. The plates were synchronised thanks to a PLL plate, whose time pulse was supplied by a GPS (Garmin 35-HVS) connected with a serial port. Digital data from four plates were passed to a commercial PC (Lippert Cool Roadrunner II) with low energy consumption, and were finally stored on a 30 Gb USB hard disk. Moreover, a 10 GB hard disc was used for the operative system and for temporary data storage (moved every 6 h to the external hard disk). Central control of the process was achieved by means of a low-power industrial PC, working under MS-DOS. The acquisition software generated raw files in a binary.ARR format. The array box was powered using 12 V batteries. The array modules used vertical L-28B Mark Products sensors as seismometers, with preamplifier and a natural frequency of 4.5 Hz. Electronic extension permitted all of the sensors to achieve a flat response curve in the 1–50 Hz frequency interval. In this way, their response was almost identical to other 1 Hz commercial sensors. Some of the systems were additionally equipped with 3-component L-4C Mark Products sensors, without preamplifiers.

*Ocean bottom seismometers (OBSs):* The OBSs used in this experiment were provided by the Lamont-Doherty Laboratory of Columbia University. These instruments have low power consumption, recording capacities exceeding 18 GB, employ 24 bit digitizers, and are equipped with broadband 3-component seismometers with noise levels below 10–16 (m/s^2^)/Hz in the band from 0.01 to 50 Hz. These ‘broadband’ seismometers are short period sensors (1 Hz geophones) coupled to efficient and very low noise amplifiers. A broadband hydrophone (differential pressure gauge) provides an additional seismo-acoustic channel. The pressure sensor is used primarily to provide a redundant channel (looking very much like vertical velocity) in case the seismic sensor fails. The pressure sensor is considered to provide good Rayleigh and body wave records, as well as good active source and micro-earthquake data at shorter periods. The instruments sample continuously at 125 Hz, and are easily converted to standard PASSCAL sampling rates. During the experiment two of the OBSs were not recovered.

*Radio-telemeters seismic stations:* In order to control background seismic activity, four analog radio-telemetered seismic stations were deployed at strategic sites on the island, with deployment designed to cover as wide a range as possible (The Spanish base with a 3-component seismometer, Fumarole Bay, Obsidians Bay, and Pendulum Cove). These stations were equipped with Mark Products L4C sensors that have a natural frequency of 1 Hz. The telemetry system consisted of a radio-frequency transmitter with a directional antenna that sent the sensor signal to the Spanish base, where it was received and recorded digitally using a de-modulator and a 16-bit A/D converter^10^.

### P-waves first arrivals

To determine P-wave first arrivals, we used the SAC routine named APK. With this algorithm, the detection of a pick is based upon abrupt changes in the ratio of a short term and long term running average of the signal. Once detected, the pick is subjected to an optional validation phase that attempts to distinguish a true event from seismic noise. Once validated, the pick is further evaluated to determine other characteristics of the event. To assess the performance of the APK routine, we compared automatically obtained picks with the real traces. Usually, the results for detected traces differed by a mean value of 0.02 s with respect to the handpicked arrivals. This can be considered an acceptable performance of the automatic routine. However, to be sure we did not use 'falsely triggered' arrivals, and to take in account as many phase arrivals as possible, we visually checked the automatic algorithm results. For this manual task, we used the UPICKER software, which permitted trace gather plotting, starting 2 s before shot time and with duration of 20 s. This choice depended on the known interval between shots, which was 120 s. We checked the error introduced by filtering the traces in SAC and found out that it is usually below 1 sample. The final database includes delay times from 70,411 crustal P-waves first arrivals automatically picked and manually checked. On average, each station recorded almost 1,500 shots.

### Nomenclature of seismic stations

In order to provide a clear nomenclature of the different seismic stations, each type of sensor received a different name. Seismic stations are summarise in Table 3 and in the excel file of the repository data with all positions.

### Multi-beam sounding

#### Multi-beam instruments

*EM120 multi-bean sounder:* To estimate the thickness of the water column below each shot, bathymetric data were collected throughout the period of the experiment using a multi-beam sounding EM120, which was able to reach up to 11.000 m b.s.l. This information was essential for producing accurate seismic tomography of the region. The main features of the multi-beam sounding EM120 are detailed in Table 4. Bathymetric profiles were collected outside of Port Foster, allowing us to obtain a high definition map of the sea floor near Deception Island volcano. The bathymetric data also revealed potential new submarine volcanic edifices and their relationships with important geodynamic characteristics^18^.

*EM-1002 multi-beam sounder:* An EM-1002 multi-beam sounder was used to collect very high precision sea floor maps. Designed to work in shallow waters (2–700 m depth), the system was operated simultaneously with the EM102 when the ‘Hesperides’ was operating inside Port Foster. Data collection stopped automatically when the depth of the seawater column was greater than 700 m. The main features of the multi-beam sounding EM-1002 are detailed in Table 5.

#### Multi-beam sounder calibration

The two legs of shooting were performed in inverse (i.e., returning back along the same path); therefore, we were able to perform fine calibrations of the multi-beam sounders. For the EM120, we identified minor errors requiring corrections for Roll (0.04°), Pitch (0.3°), and Heading (0.1°). Corrections for the EM-1002 were similar to those for the EM102, including Roll offset (−0.22°), Pitch offset (−0.7°), Heading (−0.3°), and Outer beams (0.1).

### Potential field measurements

#### SeaSPY marine magnetometer

The SeaSPY magnetometer (Marine Magnetics) is a highly sensitive/precise acquisition system and omnidirectional Overhauser sensor. This sensor, which allows measurement of the total magnetic field, remained stable for the duration of the survey period. The system has a high-precision clock (1 ppm) that can be synchronised with the onboard GPS clock. It is thermally stabilised, permitting it to operate in all type of seawater, including Antarctic waters, and owing to its design there are no heading errors. The technical features of the SeaSPY magnetometer are summarised in Table 6. The magnetometer was used during the first phase of shooting outside of Post Foster; however, its 200-m long tow cable precluded its use within the Deception Island inner bay.

#### BGM-3 marine gravimeter

The gravimeter BELL AEROSPACE-TEXTRON BGM-3 (Lockheed Martin Federal Systems) is an acquisition system that can be used with both aerial and marine vehicles. This system is composed of a gravimeter sensor mounted on a gyro-stabilised platform, and an acquisition system. Raw data are recorded and processed using a HP-486/50 computer running the BGM software, which provides two types of data, raw and previously analysed.

#### Gravimeter calibration

**Figure 6:**
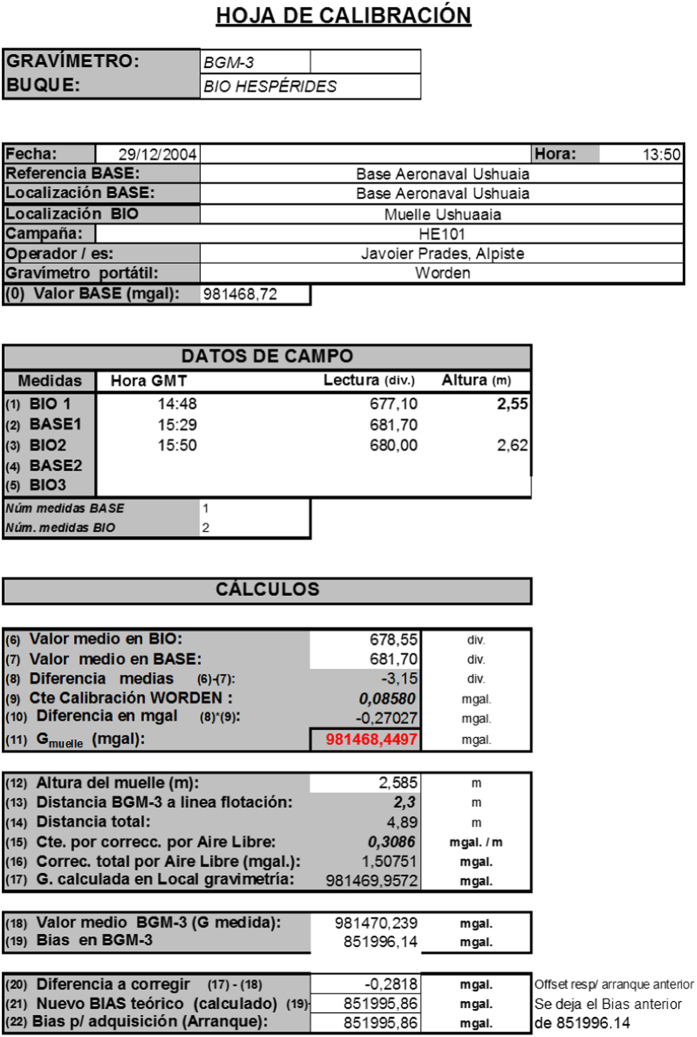


The gravimeter is regularly calibrated by the manufacturer. In addition, before every field survey, it is calibrated in the starting harbour to avoid potential shifts. The mean accuracy of the system is ±2.0 mGal. All calibration controls are performed using a WORDEN mod. MASTER portable gravimeter. In this experiment, the control was performed at the Argentinean AeroNaval (Navy and Air) base of Ushuaia on 29 December 2004, three days before the start of the field experiment. Figure 6 shows the reported calibration folder, written in Spanish, as reported by the onboard operator.

### Water measurements

In order to obtain accurate sound velocities, we also collected water-temperature profiles. The SIPPICAN MK- 21 bathy-thermo-graphic system was connected to several sensors, including temperature data (XBT sensor), sound velocity (XSV), conductivity and salinity (XCTD). This system acquires information in quasi-real time. The nominal resolution of the system is about 2% of water depth or ±0.15 °C.

## Data Records

The present database is a multidisciplinary package that contains raw data and derived results. The total size of the database is approximately 106 GB. The full data set is available from the Australian Antarctic Data Centre (AADC), which is a Public Scientific Data Repository. The AADC hosts parent metadata descriptor records that describe the whole experiment. These records are entitled ‘TOMODEC_2005_PROJECT-SPAIN’ and can be accessed from the URL ‘https://data.aad.gov.au/metadata/records/TOMODEC_2005_PROJECT-SPAIN’. The main database is connected to the parent metadata records as child metadata records with their own DOI numbers. The child data records are divided according to discipline and instrument (see table Table 7 for a complete description of all child links). Additional information on the organisation of folders and data is provided in the ‘Usage Notes’ section of this manuscript.

Continuous data from OBSs are also available in raw format from IRIS DMC (http://ds.iris.edu/gmap/XU?timewindow=2005-2005). Additional information of bathymetric data can be obtained from the Lamont-Doherty Earth Observatory IEDA Marine Geoscience Data System (http://www.marine-geo.org/). A summary of downloads that have been made of these data and documents is available from http://www.marine-geo.org/about/downloadreport/person/Ibanez_Jesus/2016A.

### Seismic data formats

**Figure 7:**
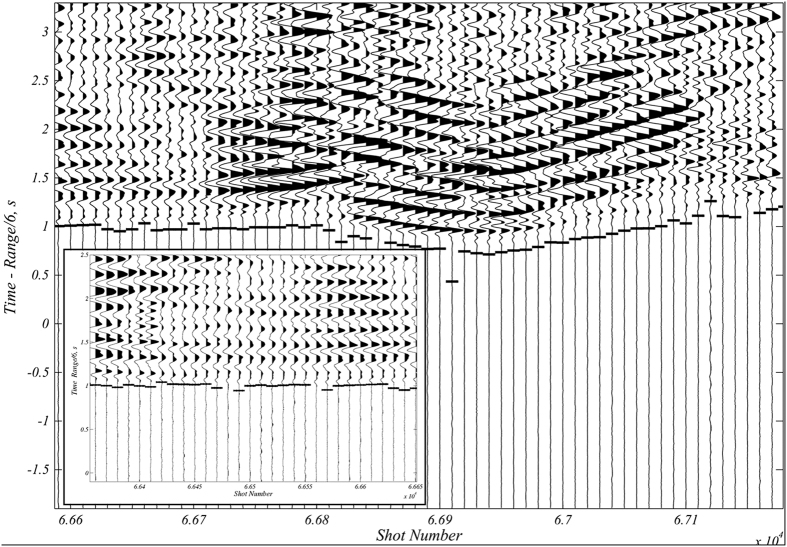
Example records for shots and their associated automatically picked first P-wave onset. The large image shows a subset of shots representing 1 in every 10. The small box contains a plot of 30 correlatives shots.

The four seismic station types (M24, Marslite, Seismic arrays, and OBSs) produced different data formats; therefore, we used different programs to handle the data. The original format of the M24 and Marslite seismic stations was binary type, but data were stored as SAC files following recovery from each station. Since the digitizer used with the M24 stations introduces a delay in the conversion from analog to digital signals, the start time of these data is always −0.2288 s before the nominal start time of the file. This was taken in account during conversion of the data to SAC format. Data from seismic array instruments were in.ARR format and were easily converted and managed using the common seismic software SEISAN, which was also used to visualise records and plot some spectral analyses. The OBS systems stored raw data in binary format organised into a file of 8,192 records. The data in each of these 1 Mb records follow a 28-byte header that contains information on the instrument, timing, and sample rate. The data were easily converted to SEGY format after retrieval. In summary, all seismic data in the database were converted to SAC and SEGY formats. The SEGY format was selected because it permits the gathering of traces in time windows after each shot and in the display of the record section. This facilitates the comparison (and identification) of waveforms. Moreover, because only 120 s of data are stored in each file, this format results in efficient manipulation and display of the data (Fig. 7).

### Seismic data quality

The quality of data registered during the seismic experiment was very good, with low noise recorded by both on-land and ocean bottom seismometers. Owing to the nature of the emplacement sites, cultural noise was completely absent. Record quality decreased for on-land stations during high winds and for OBS records when sea waves were high^23^.

**Figure 8:**
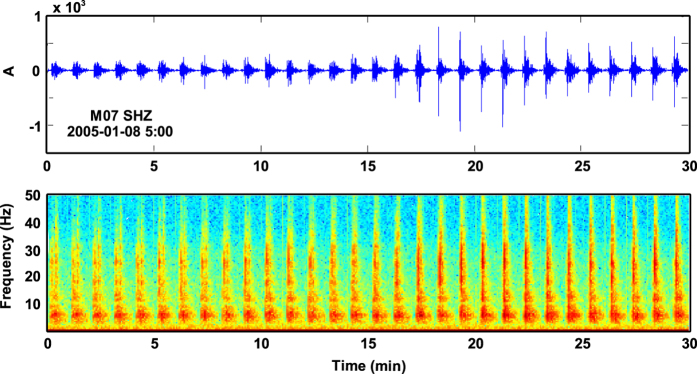


**Figure 9:**
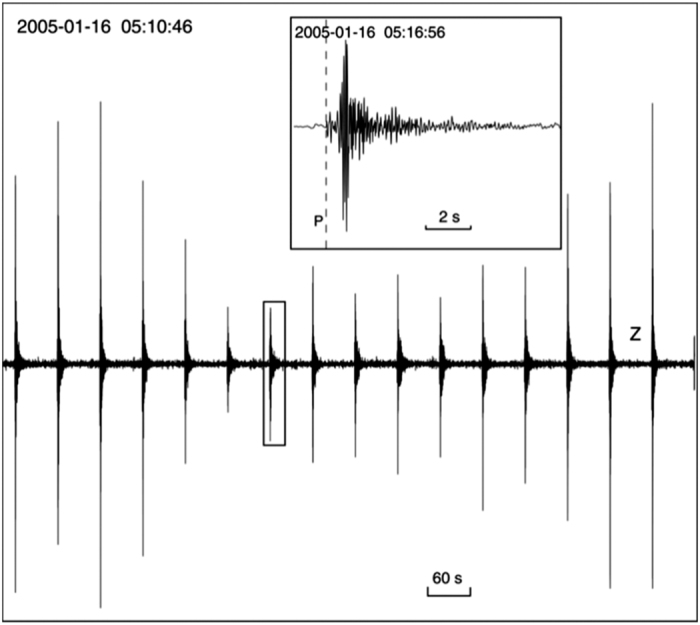


The clearest signals in seismic traces were those produced by shots (Fig. 8), although the records also included a variety of seismic signals that could be classified according to their shape, magnitude, and frequency content. For example, possible natural signals included ice-quakes, local and regional earthquakes, and tremor/long-period events. The air gun signals have P-wave arrivals from within a 40 km range. We clearly recognised crustal phases (Pg), except when they were masked by water-wave phases. These waves are easily identified owing to their travel-times (assuming a velocity of 1.5 km/s and that the shot-receiver distance is known) and to their larger amplitudes (Fig. 9). At greater distances, depending on crustal thickness, first arrivals associated with mantle refraction phases (Pn) were also identified. Interestingly, the waveforms of crustal phases with similar ray paths showed strong variability in their shapes, which was probably due to closely spaced attenuating heterogeneities in the medium (Fig. 9).

## Technical Validation

As discussed, many of the recorded data have been used to produce derived products that have been described in published manuscripts. The database contains both raw and derived data. All of the derived data are associated with seismic analyses, including P-wave first arrivals, the velocity tomographic model, 2-D scattering and an intrinsic attenuation model, and a 3-D coda-wave normalisation attenuation model. The following provides a brief description of each derived data product.

### Seismic velocity tomography

Seismic velocity tomography was the first significant output of the TOMO-DEC experiment^14^ and was performed using a well-tested method for inverting P-wave travel times^39,40^. This method applies the LSQR inversion algorithm and uses the shortest ray tracing time^40^. To obtain the 3-D tomography of Deception Island, we followed a two-step approach using two grid configurations. Firstly, we applied the method to the study of a larger region, encompassing Deception Island and surroundings. The volume, which was geometrically represented in an x, y, z Cartesian system, was 53×52×12 km wide and centred in the middle of Port Foster (62°58'S, 60°40'W). It was parameterised using 0.25-km grid-node spacing for the ray tracing and 0.5-km grid-node spacing for the perturbational grid. Secondly, we focused on Port Foster and reduced the studied area to a sub-region of 12×14×7 km, centred in the middle of the bay (62°57.2'S, 60°37.2'W). We increased the density of nodes in the parameterisation grids by using a 0.1-km grid for the ray path tracing and a 0.2 km grid for the velocity perturbation.

### Intrinsic attenuation model and 2-D scattering

**Figure 10:**
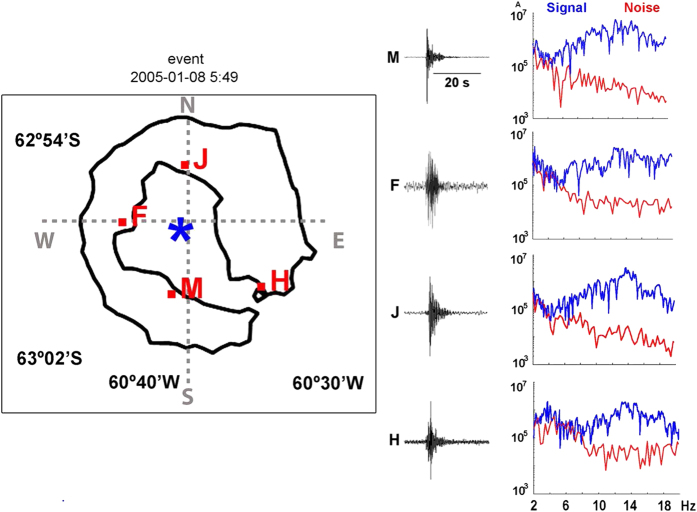
Example of the signals used to obtain the separation of the intrinsic and scattering seismic attenuation models of Deception Island. Blue denotes shot position. Red text denotes station names. To the right side is shown the signal-to-noise ratio of each seismogram as a function of frequency.

We obtained 2-D regional maps of inverse intrinsic (Qi^−1^), scattering (Qs−1), and total (Qt^−1^) quality factors for the volcanic environment of Deception Island^16^ on the basis of diffusion approximation. These maps were obtained using the above described active seismic database. Attenuation parameters were estimated by fitting observed energy envelopes to the diffusion model (Fig. 10). Then, the spatial distribution of the attenuation parameters was obtained by averaging all single source-receiver couples in 1×1 km cells. Finally, using a back-projection method based on a Gaussian-type weighting function, 2-D intrinsic- and scattering-Q images were produced. The depth for which information is obtained can be approximately estimated assuming depth equal to the minor axis^16^; in this case, 6 km on average.

### Normalisation attenuation model using 3-D coda-waves

We obtained a high-resolution 3-D P-wave attenuation tomography model using the coda normalisation method^17^, where the energy ratios of 20,293 high-quality waveforms were analysed in a single-step inversion^41^. We applied the Thurber-modified ray-bending approach^42^ in the 3-D velocity model described above. Owing to observational data associated with incoherent estimates of ray paths, the final model is restricted to depths of 1–4 km. The inverted area is a region of 20×20 km centred on Deception Island. The seismic tomography was obtained using cells of 2×2 km horizontal resolution and 1 km depth resolution with a maximum depth of 4 km b.s.l.

## Usage Notes

The TOMO-DEC experiment employed a variety of instruments and produced a variety of data types. To efficiently manage experimental information and data, the folder structure for the different metadata records deposited in the AADC is divided first according to the nature of data, and then within each folder according to instrument or derived product. The root folder, which is named TOMODEC-KNOWAVES, contains six sub-folders: SEISMIC, MAGNETIC, GRAVIMETRIC, BATHYMETRY, UTM, and MODELS (see Table 7 for complete information).

The UTM folder contains the original report provided by the science officer of the O/V ‘Hesperides’, written in Spanish and containing a description of all onboard instruments, the details of different tests, calibration, and other useful notes associated with general navigation actions.

The SEISMIC folder is subdivided according to instrument type and format. Raw data folders include: Arrays, M24, Marslite, OBS, and Telemetry. For data converted into SEGY format, folders include: SEGY-Deception, SEGY-Deception-renames, and SEGY-Deception-renames-NoOBS. Each instrument folder is composed of sub-folders associated with position. In this way, Arrays contains: A-Mekong, B-Base Argentina, C-Fumarolas, E-Bidones, F-Obsidianas, G-Pendulo, H-Balleneros, J-Crateres, K-Escondido, L-Balleneros, M-Colatinas, and N-Lobera. Similarly, M24 is subdivided in DE46, DE47, DE63, DE64, W15, W26, W27, and an additional file on their individual places. For the Marslite stations, subfolders are: W01, W04, W05, W06, W07, W10, W11, W12, W13, W14, W16, W17, W18, W23, and W25. For OBS deployed inside of Port Foster, folders include: s111, s112, s113, and s114. For OBS deployed offshore of Deception Island, folders include: s201, s202, s204, s206, s207, s208, s209, s211, s213, s301, and s302.

An excel file is provided with full information about seismometers, including type of system (arrays are named MalIAG), name of the unit, channels used, component of the seismometers (3-C or V), latitude, longitude, UTM position East and North, elevation above sea level, start and end of the recording period, and shooting phases recorded. This excel file is named stations_data_05022006.xls.

For the derived data (MODELS folder), seismic velocity tomography results for ‘Dense grid’ and ‘Small grid’ models are located in the folder ‘V models’. The associated P-wave onset database is located in the folder ‘P travel time’. Seismic attenuation results, including the bi-dimensional model separating intrinsic and scattering Q (‘Qi-Qs’) and the 3-D total Q model (‘TotalQ-3D’) are located in the folder ‘Q models’.

## Additional Information

**How to cite this article:** Ibáñez, J. M. *et al.* Database of multi-parametric geophysical data from the TOMO-DEC experiment on Deception Island, Antarctica. *Sci. Data* 4:170128 doi: 10.1038/sdata.2017.128 (2017).

**Publisher’s note:** Springer Nature remains neutral with regard to jurisdictional claims in published maps and institutional affiliations.

## Supplementary Material



## Figures and Tables

**Table 1:** Technical features of the air gun system.

**Number of air guns**	**6**
Total volume	3,520 c.i. (57.7.l)
Pick to pick pressure	50.4 bar·m (5.04 MPa, 254 db re 1 microPascal. at 1 m.)
Pick to zero pressure	21.9 bar·m (2.19 MPa, 247 db re 1 microPascal. at 1 m.)
Period of bubble in reference to the first pick	0.044 s=>22.7 Hz
Primary-bubble ratio	5.25
Maximum spectral energy	210 db
Mean spectral energy	207 db

**Table 2:** Example of shot information provided by the vessel.

**1**	**2**	**3**	**4**	**5**	**6**	**7**	**8**	**9**	**10**	**11**	**12**	**13**	**14**	**15**	**16**
10,060	30	−62.97158	−060.67955	2,005	008	02	40	22.061	8	277	54	−62.97172	−060.67710	617,756	3015,435
10,070	31	−62.97142	−060.68247	2,005	008	02	41	22.061	9	276	59	−62.97154	−060.68002	617,609	3015,460
10,080	32	−62.97102	−060.68532	2,005	008	02	42	22.061	10	287	64	−62.97135	−060.68296	617,460	3015,487
Fields: (1) Revised shot number; (2) Original shot number; (3) Ship GPS latitude (deg N); (4) Ship GPS longitude (deg E); (5) Year; (6) Julian day; (7) Hour; (8) Minute; (9) Second; (10) Navfix; (11) Ship heading; (12) Water depth (m); (13) Gun latitude (deg N); (14) Gun longitude (deg E); (15) Gun X (UTM); (16) Gun Y (UTM).															

**Table 3:** Summary of seismic stations deployed during the experiment.

**Type of seismic station**	**Name**	**Type of sensor**	**Number of sensors**
Array module	A, B, C	3-component Mark L4C	1
Array module	E, F	3-component Mark L4C and vertical L-28B	1 3-component and 8 vertical
Array module	G, H, L, M	3-component Mark L4C and vertical L-28B	1 3-component, and 9 vertical*
Array module	J	Vertical L-28B	11 vertical
Array module	K	3-component Mark L4C and vertical L-28B	1 3-component and 5 vertical in the first phase. 9 vertical in the second phase
Array module	N	Vertical L-28B	3 vertical
M24	DE46, DE47, DE63, DE64, W15, W26, W27	Lennartz LE-3D (20 s)	1
Marslite	W01, W04, W05, W06, W07, W10, W11, W12, W13, W14, W16, W17, W18, W23, W25	Lennartz LE-3Dlite MkII	1
OBS	S111, S112, S113, S114, S201, S202, S203, S204, S206, S207, S208, S209, S211, S213, S301, S302	Broadband 3-component seismometers	1
Radio-telemeter stations	FUM, OBS, PEN, BAS	3-component Mark L4C	1

*During the second phase of shooting, some vertical seismometers in arrays G and H were shifted into a smaller configuration in order to estimate the uppermost velocity structure under array sites^21^.

**Table 4:** Characteristics of multi-beam sounder EM120.

**Emission frequency**	**13 kHz**
Depth operation range	20–11,000 m
Vertical resolution	10–40 cm
Pulse length	2, 5, 15 ms
Sampling rate	2 Khz
Maximum emission rate	5 Hz
Angular coverage	150°
Number of beams	191
Size of the beam	1°×2°
Sensor	Seapath 200/MRU 5

**Table 5:** Characteristics of multi-beam sounder EM1002

**Emission frequency**	**95 kHz**
Depth operation range	2–700 m
Vertical resolution	10 cm
Pulse length	0.2, 0.7, 2 ms
Sampling rate	9 Khz
Maximum emission rate	10 Hz
Angular coverage	150°
Number of beams	111
Size of the beam	2°×2°
Sensor	Seapath 200/MRU 5

**Table 6:** Technical features of the SeaSPY magnetometer (Marine Magnetics).

**Measurement range**	**18,000–120,000 nT**
Absolute precision	0.2 nT
Sensibility of the sensor	0.01 nT
Counter sensibility	0.001 nT
Resolution	0.001 nT
Shadow region	None
Heading error	None
Temporal shift	None
Consumption	1 W stopped, 3 W maximum
Stability of temporal database	1 ppm from −45 ° C to 60 °C
Sampling rate	4 to 0.1 Hz
External trigger	Via RS-232
Communication	RS-232, 9,600 baudios
Temperature range	−45 ° C to +60 °C

**Table 7:** URL addresses, DOI for metadata and data and data citation information.

**Type of data**	**Name**	**URL**	**DOI**	**DATA CITATION**
Parent Metadata descriptor record of the whole experiment	TOMODEC_2005_PROJECT-SPAIN	https://data.aad.gov.au/metadata/records/TOMODEC_2005_PROJECT-SPAIN		
Seismic	TOMODEC_2005_SEISMOLOGY-SPAIN	https://data.aad.gov.au/metadata/records/TOMODEC_2005_SEISMOLOGY-SPAIN	doi:10.4225/15/58e6edfc522cf	Data Citation 1
Magnetic	TOMODEC_2005_MAGNETIC-SPAIN	https://data.aad.gov.au/metadata/records/TOMODEC_2005_MAGNETIC-SPAIN	doi:10.4225/15/58e47c1be64b8	Data Citation 2
Gravimetric	TOMODEC_2005_GRAVIMETRY-SPAIN	https://data.aad.gov.au/metadata/records/TOMODEC_2005_GRAVIMETRY-SPAIN	doi:10.4225/15/58e47c33f2500	Data Citation 3
Bathymetry	TOMODEC_2005_BATHYMETRY-SPAIN	https://data.aad.gov.au/metadata/records/TOMODEC_2005_BATHYMETRY-SPAIN	doi:10.4225/15/58e580cfe8c95	Data Citation 4
Additional information on onboard acquisition procedure	TOMODEC_2005_UTM-SPAIN	https://data.aad.gov.au/metadata/records/TOMODEC_2005_UTM-SPAIN	doi:10.4225/15/58e47be626ee5	Data Citation 5
Derived products	TOMODEC_2005_MODELS-SPAIN	https://data.aad.gov.au/metadata/records/TOMODEC_2005_MODELS-SPAIN	doi:10.4225/15/58e47c03e652a	Data Citation 6
